# Association between serum vitamin D status and severity of liver cirrhosis: implications for therapeutic targeting in Nigerian patients

**DOI:** 10.1186/s12876-024-03353-1

**Published:** 2024-08-12

**Authors:** Winnifred Njideka Adiri, Bruno Basil, Chinwe Philomena Onyia, Promise Asogwa, Oluchi Joy Ugwuanyi, Olive Obienu, Uchenna Nkemdilim Ijoma, Slyvester Chuks Nwokediuko

**Affiliations:** 1 Gastroenterology Unit, Department of Medicine, University of Nigeria Teaching Hospital, Ituku-Ozalla, Nigeria; 2 Department of Chemical Pathology, Benue State University, Makurdi, Nigeria; 3 Department of Medicine, Enugu State University Teaching Hospital, Parklane, Enugu, Nigeria; 4 Department of Medicine, College of Medicine, University of Nigeria, Ituku-Ozalla Campus, Enugu, Nigeria

**Keywords:** Liver cirrhosis, Nigerian, Severity, Therapeutic targeting, Vitamin D

## Abstract

**Background:**

Liver cirrhosis is a chronic and progressive liver disease with significant global health implications. Recent evidence suggests an association between serum vitamin D levels and the severity of liver cirrhosis, potentially serving as a therapeutic target. This study aimed to investigate the relationship between serum vitamin D status and the severity of liver cirrhosis in a population of Nigerian patients.

**Methods:**

This analytical, cross-sectional study involved 201 participants, including 103 with liver cirrhosis and 98 age- and sex-matched controls. Serum vitamin D was measured using ELISA, with deficiency defined as < 20 ng/ml. Cirrhosis severity was assessed using Child-Pugh and MELD scores. Spearman’s correlation was used to assess the relationship between vitamin D and severity of liver cirrhosis while ordinal regression analysis assessed its performance as an indicator of the disease severity.

**Result:**

Among cirrhotic patients, 36.9% were deficient, 31.1% insufficient, and 32.0% had sufficient vitamin D levels. Serum vitamin D showed strong negative correlations with Child-Pugh and MELD scores (*r* = -0.696, *p* < 0.001; *r* = -0.734, *p* < 0.001, respectively). Ordinal regression showed that higher vitamin D levels were associated with lower severity scores (Child-Pugh: OR = 0.856, 95% CI: 0.815–0.900, *p* < 0.001; MELD: OR = 0.875, 95% CI: 0.837–0.915, *p* < 0.001).

**Conclusion:**

Lower serum vitamin D levels correlated with increased liver cirrhosis severity, suggesting its potential as both a prognostic marker and therapeutic target. Further studies should investigate the efficacy of vitamin D supplementation in improving cirrhosis outcomes.

## Introduction

Liver cirrhosis is a chronic and progressive liver disease characterized by the replacement of healthy liver tissue with scar tissue, leading to impaired liver function [1]. It is a significant global health burden with various aetiologies, including chronic alcohol consumption, viral hepatitis, non-alcoholic fatty liver disease, and autoimmune liver diseases with hepatitis B, alcohol and self-medications being the leading causes in Nigeria [2, 3]. Despite advancements in medical care, liver cirrhosis remains a major cause of morbidity and mortality worldwide being the fourth leading cause of death and responsible for over a million deaths [4, 5]. In Nigeria, a resource-poor setting, it is a recognized cause of hospital admissions and biopsies, [6, 7] constituting a serious economic strain on patient finances as management of liver diseases is very expensive and payment for healthcare is out of pocket.

Vitamin D is a secosteroid hormone primarily known for its role in bone health and calcium metabolism [8]. However, emerging evidence suggests that vitamin D also plays crucial roles in various physiological processes beyond skeletal health, including immune modulation, inflammation regulation, cellular proliferation and differentiation among other properties [9–11]. Furthermore, vitamin D deficiency has been linked to an increased risk and progression of various chronic diseases, including liver cirrhosis [12, 13]. Vitamin D deficiency is highly prevalent in patients with chronic liver disease regardless of the aetiology, [14, 15] and is known to be associated with severe liver disease and mortality [16].

Several studies have investigated the association between serum vitamin D levels and liver cirrhosis, and many have reported lower serum vitamin D levels in patients with liver cirrhosis compared to healthy individuals [16–20]. There is evidence suggesting a bidirectional association between liver cirrhosis and vitamin D deficiency. Mechanisms via which liver cirrhosis can lead to the deficiency of vitamin D include impairment of hepatic synthesis and metabolism, malabsorption due to impaired bile acid synthesis, and decreased exposure to sunlight due to fatigue and limited mobility [11, 12]. On the other hand, Vitamin D deficiency may worsen liver cirrhosis by dysregulation of immune responses, increased insulin resistance and metabolic dysfunction contributing to the progression of liver steatosis, fibrosis and cirrhosis, and impairment of liver regeneration [21–23]. This forms the basis of our hypothesis that serum vitamin D levels are closely associated with liver cirrhosis, may be a valuable indicator of its severity and serve as a potential therapeutic target. As such, there may be a need to assess, with the intention to augment, vitamin D levels in patients with chronic liver diseases, who are also deficient in the vitamin, as a means of forestalling progression to and worsening of liver cirrhosis.

Determining the severity of liver cirrhosis is an important step in the management of the disease, and its accurate assessment is most reliable by biopsy, for which assessment of the severity of cirrhosis is not a justifiable indication considering its cost, invasiveness and risk of complications, [24] hence the need for other surrogate markers. Various noninvasive tests, biomarkers and scoring systems have been evaluated for this purpose with varying diagnostic performances [25]. For instance, the Child-Pugh and Model for End-Stage Liver Disease (MELD) scoring systems have proven effective for severity classification, however, these do not serve as potential therapeutic targets in the management of liver cirrhosis. This study aims to investigate the relationship between serum vitamin D levels, a potential therapeutic target, and the severity of liver cirrhosis in the study population. By correlating vitamin D status with established markers of cirrhosis severity, we aim to examine the potential of serum vitamin D as an index of severity of liver cirrhosis with possible therapeutic implications amongst Nigerian patients.

## Methods

### Study design and setting

This study was conducted between January 2021 and January 2022 (13 months) and employed an analytical non-interventional cross-sectional and controlled design to investigate the relationship between serum Vitamin D levels and the severity of liver cirrhosis in a population of Nigerian patients with liver diseases attending the medical out-patient clinic (MOPD) at UNTH. It was conducted in compliance with the Declaration of Helsinki [26]. Number codes were allotted to each recruited participant, and their clinical data and test results were locked out in secured spaces to ensure confidentiality throughout the study.

The sample size was determined using a prevalence of liver cirrhosis among hospital patients with liver diseases, 20.4% [6], a margin of error of 0.082, and a confidence interval of 95%. Using the formula for calculating sample size in cross-sectional studies, [27] and adjusting for a 10% non-response rate, a minimum sample size of 101 was determined to be necessary for this study.

Participants were adult patients diagnosed with liver cirrhosis, irrespective of the aetiology, and individuals without any liver-related diseases served as age- and sex-matched controls. Patients that were less than 18 years, pregnant or lactating, or with other major comorbid conditions which may alter vitamin D balance including renal failure, malabsorption and hypoparathyroidism, or on drugs like corticosteroids, anti-epileptic drugs, etc., or anti-viral chemotherapy for Hepatitis B virus (HBV), Hepatitis C virus (HCV) and Human immunodeficiency virus (HIV) infection, were all excluded to ensure the homogeneity of the study group.

### Data collection

Patient bio-data and medical history were obtained by the use of a validated and structured research proforma. Variables of interest included demographics (age, gender), anthropometrical measurements (weight and height), clinical parameters (aetiology and duration of liver cirrhosis, presence of liver-related complications, the severity of liver cirrhosis, etc.), laboratory measurements (serum levels of vitamin D (25-hydroxyvitamin D)), liver function tests e.g., serum bilirubin, albumin, international normalized ratio (INR), full blood count (FBC), renal function tests e.g., serum creatinine, markers of fibrosis e.g., serum levels of alpha-fetoprotein (AFP). In addition to comprehensive clinical and laboratory assessment, abdominal ultrasound was utilized to assess liver morphology, including the presence of nodularity, irregularity of the liver surface, and signs of portal hypertension such as splenomegaly and portosystemic collaterals. Additionally, liver histology obtained through percutaneous liver biopsy demonstrating architectural distortion, fibrosis, and regenerative nodules provided definitive confirmation of the diagnosis where available. A combination of clinical features, abdominal ultrasound, and liver histology were used to make the diagnosis of liver cirrhosis.

### Assessment of severity of liver cirrhosis

Liver cirrhosis severity was evaluated using established scoring systems, including the Child-Pugh classification and the Model for End-Stage Liver Disease (MELD) score. The Child-Pugh score was calculated based on serum bilirubin, serum albumin, prothrombin time or international normalized ratio (INR), presence and severity of ascites, and presence and severity of hepatic encephalopathy were used to determine severity scores ranging from 5 to 15 and to classify them into Child-Pugh classes A, B, or C based on severity of liver cirrhosis. Also, the MELD score (MELD = 3.78 × ln[serum bilirubin (mg/dL)) + 11.2 × ln(INR) + 9.57 × ln(serum creatinine 9 mg/dL)) + 6.43) calculated using serum levels of bilirubin, creatinine, and the international normalized ratio (INR) was used to classify them into 5 groups denoted as < 9, 10–19, 20–29, 30–39 and ≥ 40 based on their estimated 3 months mortality [28].

### Serum vitamin D measurement

Five millilitres (5 mL) of venous blood samples were drawn from the median cubital vein into plain vacutainer tubes while maintaining aseptic measures. The samples were left to stand until clotting and retraction had occurred, then centrifuged for 10 min at the speed of 2000–3000 rpm. Then, the supernatant was collected and stored at -20^o^C until analysis which was done in batches within one month. Serum Vitamin D levels in nanograms per millilitre (ng/mL) were assayed using quantitative sandwich enzyme-linked immunosorbent assay (ELISA) technique. The assay kits were provided by Calbiotech, 1935 Cordell Court, El Cajon, California, USA. All other laboratory tests including liver function tests, renal function tests and hematological testing were conducted using routine laboratory testing methods.

Vitamin D deficiency was defined as 25(OH)D levels < 20 ng/ml, values between 20 and 30 ng/mL and 30–100 ng/mL were defined as vitamin D insufficiency and sufficiency respectively, while values between 30–50ng/ml are considered optimal [12].

### Statistical analysis

Statistical analysis was performed using the Statistical Package for Social Sciences (SPSS) computer software version 25.0 for Windows (IBM, Inc, USA). Descriptive statistics, including means, standard deviations, and frequencies, were used to summarize quantitative and qualitative variables. Univariate analysis was performed to compare the means of continuous independent variables like age, BMI, etc., in participants with and without Vitamin D deficiency using the independent T-test, while the Chi-square test was used for categorical independent variables like sex, educational status, etc. Tests of normality were conducted on the data using the Shapiro-Wilk test to determine whether the variables of interest were normally distributed, and a value less than 0.05 indicated that the data was not normally distributed.

An analysis of variance (ANOVA) was performed to compare mean serum Vitamin D levels across different liver cirrhosis severity categories, and correlations between Vitamin D levels and severity were assessed using Spearman’s correlation coefficients for non-parametric distribution. The performance of serum Vitamin D level as an indicator of liver cirrhosis severity was determined by ordinal regression analysis while controlling for confounding factors like BMI. The model fitting information and goodness-of-fit test were used for the calibration of the model and values less than 0.05 and greater than 0.05 respectively, indicated a good fit. Statistical significance was considered to be at a probability value of < 0.05.

## Results

A total of 201 individuals participated in this study, of whom 103 had been diagnosed with liver cirrhosis while 98 served as healthy age and sex-matched controls. The baseline socio-demographic and clinical characteristics of the study participants are presented in Table 1. Statistically significant differences were noted only in the consumption of significant amounts of alcohol (males > 40 g/day, females > 20 g/day) in 9.2% (*n* = 9) of the control group versus 39.8% (*n* = 41) of the liver cirrhosis group (*p* = 0.031), and BMI which was higher in the liver cirrhosis group (0.7 ± 6.3 kg/m^2^ versus 33.9 ± 4.3 kg/m^2^; *p* = 0.017).

**Table 1:** Baseline socio-demographic and clinical characteristics of the study participants

Participant Characteristics	Control group (*N* = 98) Mean ± SD or *n* (%)	Liver Cirrhosis group (*N* = 103) Mean ± SD or *n* (%)	*p*-Value
Age (years)	47.3 ± 7.8	46.8 ± 9.2	0.178
Sex:			0.567
*Male* *Female*	73 (74.5) 25 (25.5)	76 (73.8) 27 (26.2)	
Occupation:			0.124
*Unemployed* *Traders* *Civil Servants* *Farmers* *Others*	13 (13.3) 40 (40.8) 15 (15.3) 23 (23.5) 7 (7.1)	15 (14.6) 38 (36.9) 17 (16.5) 22 (21.4) 11 (10.7)	
Educational status:			0.301
*Primary* *Secondary* *Tertiary*	26 (26.5) 35 (35.7) 37 (37.8)	29 (28.2) 33 (32.0) 41 (39.8)	
Marital Status:			0.067
*Single* *Married* *Divorced*	27 (27.6) 66 (67.3) 5 (5.1)	32 (31.1) 68 (66.0) 3 (2.9)	
Alcohol Consumption:			0.031*
*Male: > 40 g/day* *< 40 g/day* *Nil*	9 (9.2) 30 (30.6) 34 (34.7)	39 (37.9) 20 (19.4) 17 (16.5)	
*Female: > 20 g/day* *< 20 g/day* *Nil*	0 (0.0) 3 (3.1) 22 (22.5)	2 (1.9) 6 (5.8) 19 (18.4)	
Smoking:			0.987
*No* *Yes*	84 (85.7) 14 (14.3)	88 (85.4) 15 (14.6)	
BMI (kg/m^2^)	28.7 ± 6.3	33.9 ± 4.3	0.017*
Co-morbidities:			0.520
*Hypertension* *Diabetes mellitus* *Diabetes/Hypertension* *Others*	15 (15.3) 16 (16.3) 12 (12.2) 3 (3.1)	19 (18.4) 26 (25.2) 18 (17.5) 5 (4.9)	

*p-value significant at < 0.05; N = Number of observations; SD = Standard Deviation

Furthermore, Table 2 presents the results of laboratory investigations of the two groups of participants. Statistically significant differences were seen in the liver cirrhosis group versus the control group with regards to the mean values of ESR (male 42.4 ± 35.0 mm/hr; female 50.0 ± 41.2 mm/hr versus male 32.6 ± 17.4 mm/hr; female 37.0 ± 19.8 mm/hr; *p* = 0.011), albumin (26.5 ± 9.0 g/L versus 41.7 ± 6.5 g/L; *p* < 0.001), globulin (41.2 ± 9.1 g/L versus 32.1 ± 5.5 g/L; *p* = 0.011), urea (3.9 ± 2.5 mmol/L versus 7.4 ± 2.1 mmol/L; *p* < 0.001), total bilirubin (82.9 ± 85.8 µmol/L versus 17.1 ± 9.2 µmol/L; *p* = 0.021), conjugated bilirubin (57.5 ± 65.7 µmol/L versus 7.3 ± 4.1 µmol/L; *p* = 0.033), serum vitamin D level (25.7 ± 13.0 ng/mL versus 44.6 ± 14.8 ng/mL; *p* < 0.001), and HBsAg positivity in 76.6% (*n* = 82) versus 11.2% (*n* = 11)(p,0.001) respectively. However, no significant differences in other laboratory parameters were noted.

**Table 2:** Baseline laboratory investigations of the study participants

Participant Laboratory Investigations	Control group (*N* = 98) Mean ± SD or *n* (%)	Liver Cirrhosis group (*N* = 103) Mean ± SD or *n* (%)	*p*-Value
Hb (g/dL):			0.190
*Male* *Female*	10.8 ± 1.6 9.6 ± 1.2	11.2 ± 2.0 10.9 ± 1.4	
Platelets (x10^9^/L)	161.1 ± 63.8	159.2 ± 67.3	0.213
White blood cells (10^9^/L)	6.8 ± 1.8	7.4 ± 1.9	0.340
INR	1.5 ± 0.9	1.6 ± 0.6	0.511
ESR (mm/hr):			0.011*
*Male* *Female*	32.6 ± 17.4 37.0 ± 19.8	42.4 ± 35.0 50.0 ± 41.2	
FPG (mmol/L)	5.1 ± 1.5	4.9 ± 1.8	0.103
Albumin (g/L)	41.7 ± 6.5	26.5 ± 9.0	< 0.001*
Globulin (g/L)	32.1 ± 5.5	41.2 ± 9.1	0.011*
Creatinine (µmol/L)	98.9 ± 45.3	104.3 ± 68.9	0.121
Urea (mmol/L)	7.4 ± 2.1	3.9 ± 2.5	< 0.001*
Calcium (mmol/L)	2.4 ± 0.3	2.4 ± 0.1	0.452
Phosphorus (mmol/L)	1.2 ± 0.5	1.3 ± 0.2	0.268
Total Bilirubin (µmol/L)	17.1 ± 9.2	82.9 ± 85.8	0.021*
Conjugated Bilirubin (µmol/L)	7.3 ± 4.1	57.5 ± 65.7	0.033*
AST (U/L)	55.1 ± 55.5	69.0 ± 72.9	0.052
ALT (U/L)	31.0 ± 19.2	29.4 ± 17.3	0.301
ALP (U/L)	108.8 ± 56.3	115.2 ± 77.5	0.091
Alpha-fetoprotein (ng/ml)	10.7 ± 1.8	12.2 ± 2.4	0.059
Serum 25(OH)D (ng/mL)	44.6 ± 14.8	25.7 ± 13.0	< 0.001*
HBsAg:			< 0.001*
*Negative* *Positive*	87 (88.8) 11 (11.2)	21 (20.4) 82 (79.6)	
Anti-HCV:			0.333
*Negative* *Positive*	98 (100.0) 0 (0.0)	100 (97.1) 3 (2.9)	

*p-value significant at < 0.05; N = Number of observations; SD = Standard Deviation

**Fig. 1:**
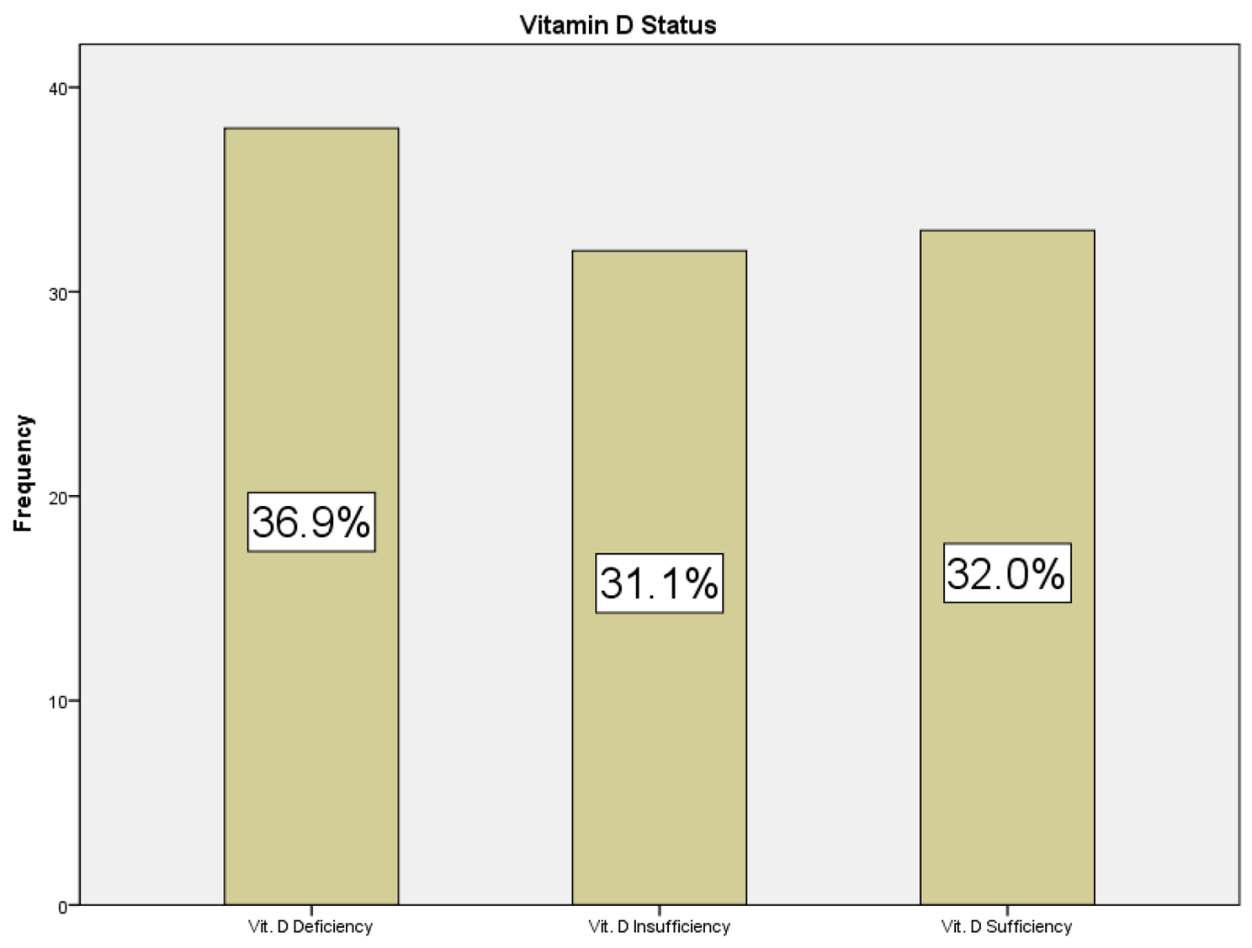
Distribution of vitamin D status amongst participants with liver cirrhosis

The pattern of distribution of participants with liver cirrhosis based on their vitamin D status indicates that 36.9% (*n* = 38) were vitamin D deficient, 31.1% (*n* = 32) had vitamin D insufficiency while 32.0% (*n* = 33) had sufficient serum levels of the hormone (Fig. 1).

**Fig. 2:**
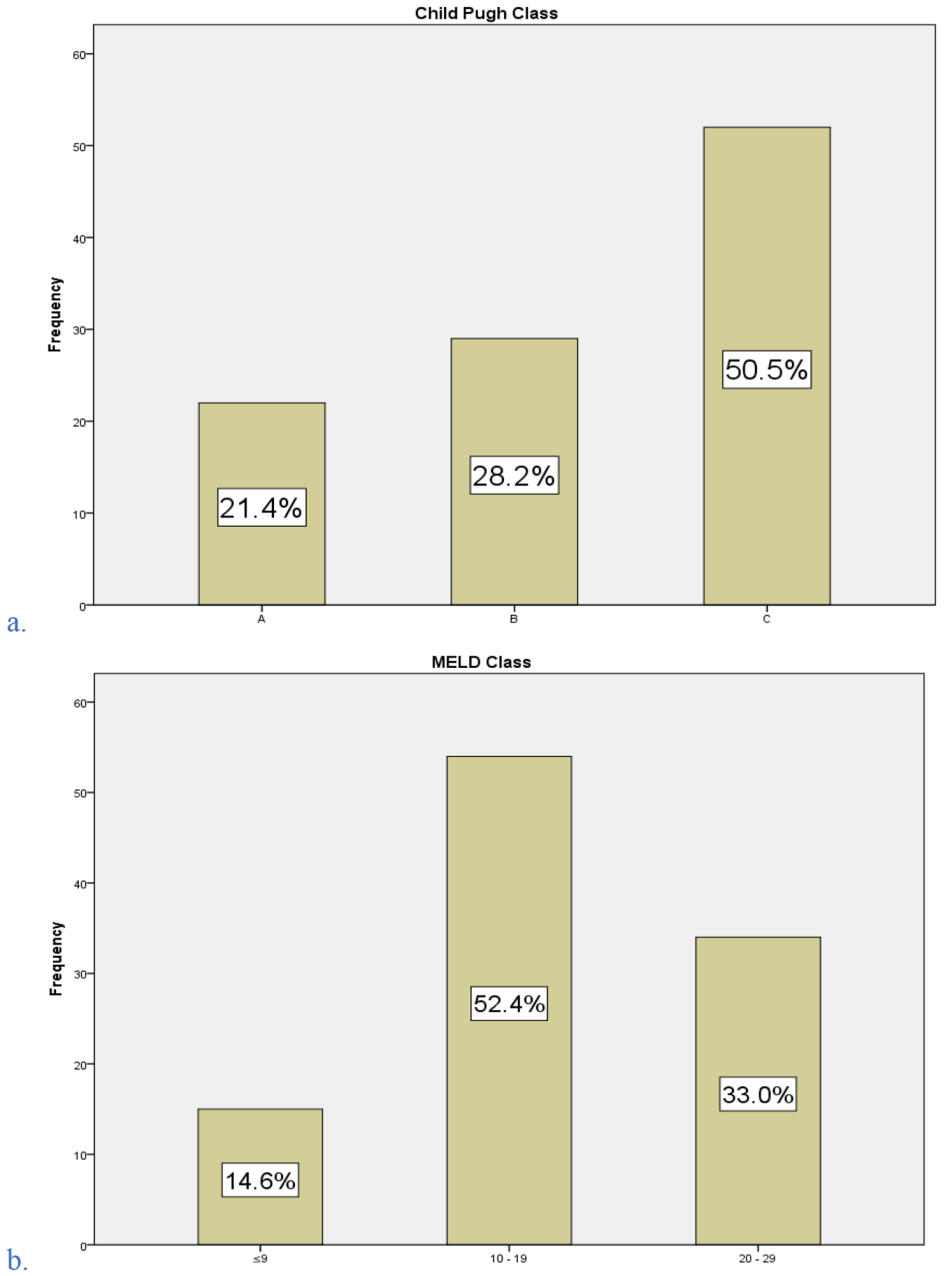
Distribution of participants with liver cirrhosis based on severity classifications

With regards to the severity of liver cirrhosis, Child-Pugh class C accounted for the most, 50.5% (*n* = 52), of all cirrhotic participants while the majority, 52.4% (*n* = 54), were of the “10–19” category in the MELD class based on 3-months risk of mortality. A notable finding is the absence of degrees of severity greater than the “20–29” category on the MELD classification. (Fig. 2).

**Fig. 3:**
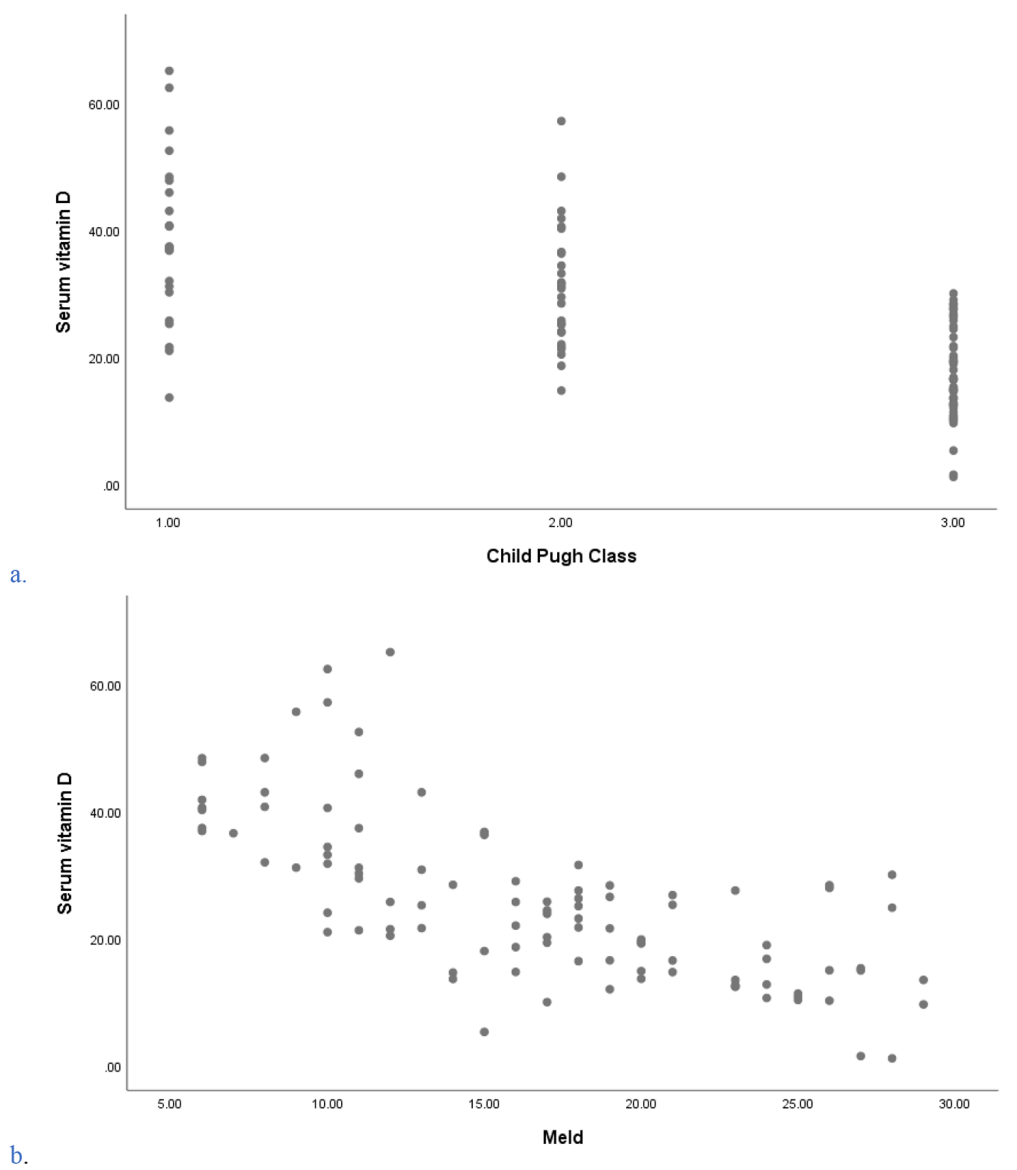
Scatter plot showing the correlation between serum vitamin D and severity of Liver cirrhosis

The correlation between serum vitamin D levels and the severity of liver cirrhosis based on CPS and MELD Scores was assessed. After controlling for potential confounders like BMI and alcohol consumption, serum vitamin D had strong negative correlations with CPS (*r* = -0.696, p = < 0.001) and MELD score (*r* = -0.734, p = < 0.001) (Fig. 3). This correlation is evident in Table 3 which shows the statistically significant relationship between the severity of cirrhosis based on the CPS and MELD classes versus the mean serum vitamin D levels (*p* < 0.001).

**Table 3:** Comparison of serum vitamin D level and severity of liver cirrhosis

Severity Category	Liver Cirrhosis (*N* = 103) *n* (%)	Serum 25(OH)D (ng/mL) Mean ± SD	*p*-value
Child-Pugh:			
Class A Class B Class C	22 (21.3) 29 (28.2) 52 (50.5)	38.6 ± 13.4 30.8 ± 9.5 17.4 ± 7.2	< 0.001*
MELD Class:			
≤ 9 10–19 20–29	15 (14.6) 54 (52.4) 34 (33.0)	40.7 ± 7.1 27.7 ± 12.1 16.0 ± 7.1	< 0.001*

*p-value significant at < 0.05; N = Number of observations; SD = Standard Deviation

The assessment of serum vitamin D levels as an indicator of disease severity in patients with liver cirrhosis was carried out using ordinal regression analysis. It shows that an increase in serum vitamin D level was associated with a decrease in the odds of higher severity category by an odds ratio of 0.856 (95% CI: 0.815–0.900, *p* < 0.001) for the Child-Pugh score, and 0.875 (95% CI: 0.837–0.915, *p* < 0.001) for the MELD score.

## Discussion

In this study, possible associations were observed between liver cirrhosis and both significant alcohol consumption and a history of hepatitis B viral infection. These factors are associated with alcoholic liver disease and chronic inflammatory response mechanisms respectively, and are consistent with their established role as leading causes of the disease globally and particularly in Nigeria [2, 3, 29]. Also, the elevated BMI in the liver cirrhosis group aligns with previous findings suggesting obesity as a risk factor for non-alcoholic fatty liver disease, a common precursor to liver cirrhosis [30]. Other significant laboratory findings in the liver cirrhosis group include the increased ESR indicative of chronic inflammation and tissue damage, altered albumin and globulin levels reflecting impairment of hepatic synthetic function and immune dysregulation, lower urea levels due to decreased synthesis, and elevated bilirubin levels consistent with hepatocellular dysfunction and cholestasis. These findings highlight the complex pathophysiology of liver cirrhosis and the importance of these biomarkers in its diagnosis and management, as seen in previous studies [31–34].

There was a high prevalence of vitamin D deficiency among liver cirrhosis patients in the studied population, as has been previously reported [12–16, 35, 36]. Majority of participants were classified as Child-Pugh class C and MELD score category 10–19, indicating an increased likelihood of advanced disease stages. Notably, there was an absence of participants classified in severity categories greater than MELD score 20–29, suggesting a possible ceiling effect in the MELD score classification system for this study population. This finding may reflect the limited sample size or certain characteristics of the study population, warranting further investigation. The high burden of advanced cirrhosis among liver cirrhosis individuals with vitamin D deficiency or insufficiency may suggest the need for targeted interventions to improve vitamin D status and potentially mitigate disease progression.

Furthermore, the significant and strongly negative correlations between serum vitamin D level and liver cirrhosis severity in this study indicate that higher levels are associated with less severe cirrhosis, suggesting the need for further longitudinal studies to assess the potential role of vitamin D in modulating disease progression. This inverse relationship is consistent with previous studies with similar findings [21–23]. The high prevalence of vitamin D deficiency despite the presence of ample sunlight in this Nigerian population suggests that other non-metabolic factors beyond sunlight exposure and liver disease influence vitamin D status and its impact on liver cirrhosis. These factors may include dietary habits, cultural practices, and dark skin colour, all of which can affect vitamin D absorption, conversion, and utilization [37, 38]. Its observed association with liver cirrhosis suggests that optimizing vitamin D status may warrant further investigation for potential therapeutic implications in managing this condition. Vitamin D has been shown to possess anti-inflammatory, anti-fibrotic, and immunomodulatory properties, all of which are relevant to the pathogenesis and progression of liver cirrhosis [12, 39].

Studies have demonstrated that vitamin D supplementation can improve liver function tests, reduce hepatic inflammation and fibrosis, decrease the risk of hepatocellular carcinoma, and improve functional status assessed by the Child-Pugh scale in patients with chronic liver disease [40, 41]. However, further research is required to explore the clinical implications of vitamin D supplementation in liver cirrhosis, evaluate the efficacy and safety as an adjunctive therapy and delineate the details of its use including threshold for initiating therapy, dosage, optimal duration, delivery routes and therapeutic monitoring. These are necessary considerations when tailoring its use to various populations.

Some clinical applications of serum vitamin D assay have been suggested, such as its utilization as a non-invasive marker of liver fibrosis in chronic hepatitis C, as a prognostic factor for infections and death in individuals with liver cirrhosis, and as a marker of poor prognosis in patients with hepatocellular carcinoma [12]. In this study, the potential role of serum vitamin D as an indicator of the severity of liver cirrhosis was explored using ordinal regression analysis. It showed significant associations between increasing serum vitamin D levels and decreased odds of higher severity category for both Child-Pugh and MELD scores, supporting its predictive value as a marker of disease severity classification.

There are a few limitations to the index study. Firstly, the cross-sectional nature of our study limits our ability to establish causality between serum vitamin D levels and the severity of liver cirrhosis. Longitudinal and interventional studies are needed to validate these findings and elucidate the mechanistic pathways underlying the observed associations. Additionally, while our study provides valuable insights into the association between serum vitamin D levels and the severity of liver cirrhosis, it does not assess the therapeutic effects of vitamin D supplementation. Further studies using Randomized Controlled Trials would be required to determine whether vitamin D supplementation can significantly improve outcomes in liver cirrhosis patients and evaluate its performance in light of the geographical, cultural and constitutional factors that may affect serum vitamin D in the Nigerian population. Finally, the findings of this study are based on a group of participants from a specific geographic region, which may limit the generalizability of the results to other populations. Future research should aim to validate these findings in diverse settings to assess their broader relevance.

## Conclusion

The significant strong negative correlation observed between serum vitamin D levels and liver cirrhosis severity in this study suggests that higher vitamin D levels are associated with less severe cirrhosis. While these findings highlight the importance of vitamin D status in patients with liver cirrhosis, further studies are necessary to explore the potential therapeutic role of vitamin D in modulating disease progression and outcomes.

## Data Availability

The datasets from this study will be available upon reasonable request to the corresponding author. This is because the dataset includes additional data irrelevant to this study and may require exclusion.

## Abbreviations

UNTH: University of Nigeria Teaching Hospital
BMI: Body Mass Index
ESR: Erythrocyte Sedimentation Rate
HBV: Hepatitis B Virus
HCV: Hepatitis C Virus
HIV: Human Immunodeficiency Virus
FBC: Full Blood Count
AFP: Alpha–Fetoprotein
ELISA: Enzyme–Linked Immunosorbent Assay
SPSS: Statistical Package for Social Sciences
INR: International Normalized Ratio
ANOVA: Analysis of Variance
CI: Confidence Interval
CPS: Child–Pugh Score
MELD: Model for End–Stage Liver Disease
HBsAg: Hepatitis B Surface Antigen
T: test–Student’s t–test
IQR: Interquartile Range
